# Honeysuckle-derived microRNA2911 inhibits tumor growth by targeting TGF-β1

**DOI:** 10.1186/s13020-021-00453-y

**Published:** 2021-06-29

**Authors:** Chunyan Liu, Mengzhen Xu, Luocheng Yan, Yulian Wang, Zhen Zhou, Shaocong Wang, Yajie Sun, Junfeng Zhang, Lei Dong

**Affiliations:** State Key Laboratory of Pharmaceutical Biotechnology, School of Life Sciences, Nanjing University, 163 Xianlin Avenue, Nanjing, 210093 China

**Keywords:** Honeysuckle, miR2911, Colon cancer, TGF-β1

## Abstract

**Background:**

Honeysuckle is a time‐honored herb with anticancer activity in traditional Chinese medicine. Recently, accumulating reports are suggesting that the microRNAs in this medicinal plant not only play a physiological role in their original system, but also can be transmitted to another species as potential therapeutic components. In the numerous bioactive investigations, the anti-tumor effects of these microRNAs in the magical herb are rarely studied, especially the special miR2911, a honeysuckle-encoded atypical microRNA, with high stability during the boiling process and unique biological activity to target TGF-β1 mRNA.

**Methods:**

Luciferase assay was conducted to test the ability of miR2911 to target TGF-β1 mRNA. ELISA was performed to determine the expression level of TGF-β1 of mouse colorectal adenocarcinoma CT26 cells when treated with miR2911 and tumor tissue in *Sidt1*^+*/*+^ and *Sidt1*^*−/−*^ mice. qRT-PCR was performed to examine the level of expression of miR2911. Tumor-bearing wild and nude mice were employed to evaluate the anti-tumor effect of honeysuckle and miR2911 in vivo. Tumor tissue necrosis was observed by H&E staining. Besides, the infiltration of T lymphocytes across solid tumors was tested by immunostaining staining.

**Results:**

Our results showed that honeysuckle slowed the development of colon cancer down. Further research showed that miR2911 could bind strongly to TGF-β1 mRNA and down-regulate the expression of TGF-β1 and had a high stability under boiling and acid condition. Moreover, SIDT1 mediated dietary miR2911 inter-species absorption. And we found that miR2911 had a similar anticancer effect as honeysuckle. Mechanistically, miR2911 reversed the tumor-promoting effect of TGF-β1 by an increase of T lymphocytes infiltration, resulting in slowing the colon cancer process in immunocompetent mice. Consistent with this inference, the anti-tumor effect of miR2911 was revealed to be abolished in T cell immune deficiency mice.

**Conclusion:**

Taken together, honeysuckle-derived miR2911 showed an anti-tumor effect in colon cancer through targeting TGF-β1 mRNA. The down-regulation of TGF-β1 promoted T lymphocytes infiltration, and accordingly impeded the colon tumor development.

**Supplementary Information:**

The online version contains supplementary material available at 10.1186/s13020-021-00453-y.

## Background

Honeysuckle is a famous traditional Chinese medicine owing to its powerful anti-inflammation, anti-oxidation, anti-microbial, anti-virus and anti-tumor pharmacological properties [1–4]. The aqueous extract of honeysuckle can kill cancer cells in vitro and decrease solid tumor volumes in vivo [5, 6]. Honeysuckle contains abundant compounds with anticancer activity, and the related mechanisms have been widely studied [7–9]. Honeysuckle has a wide variety of microRNAs, including miR166g, miR2914, miR2910, and miR2911 [10]. These microRNAs have been reported to control various biological processes by binding to the 3′ UTR of target mRNA transcripts to facilitate their degradation and/or inhibit their translation [11–13]. Interestingly, some target genes of honeysuckle-derived microRNAs play important roles in cancer development and formation of tumor environment.

Since fundamental questions are still unanswered-how these microRNAs transport from plants to mammals-whether microRNAs are involved in the anti-tumor effects of honeysuckle is rarely studied. A recent study has shown that miR2911 could realize the cross-kingdom transfer via stomach-enriched SID-1 transmembrane family member 1 (SIDT1) transporter [14]. The presence of the transfer mechanism suggests that the anti-tumor effect of honeysuckle-derived microRNAs is worthy of study. Our previous study revealed that miR2911 is a special microRNA in honeysuckle due to its high stability during the boiling process [10]. Meanwhile, miR2911 could target 3′ UTR of transforming growth factor-β1 (TGF-β1) mRNA transcripts to decrease the TGF-β1 expression, resulting in relieving liver fibrosis in the model of CCl_4_-induced liver fibrosis [14]. TGF-β1 is a key homeostatic factor that balances the immune system [15–17]. Previous studies have shown that TGF-β1 could inhibit the maturation and differentiation of bone marrow-derived T cells [18–20]. Meanwhile, activation of TGF-β1 signaling pathways inhibited T cell activation and infiltration in tumors [21–23]. This means that TGF-β1 is not only a potential therapeutic target for fibrosis, but also a target of miR2911 for anticancer therapy.

In the present study, we intended to evaluate the anti-tumor effects of miR2911 through a series of systematic analyses. In particular, its restriction on the functionality of TGF-β1 is the focus of this study. Besides, we expected the interference effects of miR2911 on TGF-β1 could subsequently regulate the local immunological environment and lead to increased mobilization of T lymphocytes in tumor tissue for restraining the colon cancer development.

## Methods

### Honeysuckle

Honeysuckle was bought from a Chinese herbal medicine shop (Nanjing, China). Honeysuckle was prepared by boiling 10 g of honeysuckle in 100 mL water for 30 min, resulting in ~ 50 mL decoction.

### Boiling treatment and low pH treatment

To verify the stable existence of honeysuckle-derived miR2911 under prolonged boiling operation and acidic conditions, we treated it with boiling and acid. 10 g of honeysuckle was added to 100 mL water and boiled in a high fire. Then the fire turned smaller and continued to cook the honeysuckle for 30 min. After filtration, 50 mL of honeysuckle decoction was obtained. Keep boiling the decoction and the miR2911 level was detected at 0 h, 1 h, 2 h and 3 h. For low pH treatment, honeysuckle decoction was acidified by HCl to pH 2.0 and the miR2911 level was detected after treating with acid for 0 h, 1 h, 2 h and 3 h.

### RNA isolation and quantitative real-time polymerase chain reaction (qRT-PCR)

To calculate the level of expression of miR2911 and other microRNAs in honeysuckle, total RNA from honeysuckle and honeysuckle decoction were extracted using the Universal Plant MicroRNA Kit (Bioteke, Beijing, China). qRT-PCR was performed using TaqMan microRNA probes (Applied Biosystems, Froster City, CA, USA) according to the manufacturer’s instructions.

### miR2911 and ncRNA

All synthetic microRNAs were customized from TaKaRa (Dalian, China). miR2911 (5′-ggccgggggacgggcuggga-3′) and ncRNA (5′-uuguacuacacaaaaguacug-3′) were synthesized by GenePharma (Shanghai, China).

### Animal

C57BL/6 and nude BALB/c mice were purchased from Vital River Laboratories (Beijing, China). *Sidt1*^*−/*−^ mice (strain Sidt1tm1(KOMP)Vlcg) and *Sidt1*^+*/*+^ mice were obtained from the Knockout Mouse Project (KOMP) Repository at University of California, Davis (CA, USA). The animals were fed in a specific pathogen-free (SPF) animal facility with controlled light (12 h light/dark), temperature and humidity, with food and water available. Animal protocols were reviewed and approved by the Animal Care and Use Committee of Nanjing University, and conformed to the Guidelines for the Care and Use of Laboratory Animals published by the National Institutes of Health.

### Cell culture

Mouse colorectal adenocarcinoma cells (CT26) were obtained from Institute of Biochemistry and Cell Biology (Shanghai, China). CT26 cells were cultured in Roswell Park Memorial Institute-1640 medium (RPMI-1640, Thermo Fisher Scientific, Waltham, MA, USA) containing 10% (v/v) fetal bovine serum (FBS, Hyclone, Logan, UT, USA). Cultures were maintained at 37 °C in a CO_2_ incubator.

### Establish of tumor-bearing mice model and treatment protocol

The mice were axillary implanted CT26 cells. The mice were daily gavage fed with 0.5 mL honeysuckle decoction or 0.1 nmol miR2911 1 week after CT26 cells transplant. The diameters of the tumors and survival rate of the mice were recorded every week. In 8th week, the mice were sacrificed and the tumors were collected for analysis. For *Sidt1*^+*/*+^ and *Sidt1*^*−/*−^ mice, they were given the synthetic miR2911 (0.1 nmol) orally or intravenously on a daily basis after the tumor-bearing mice model was established. Also in 8th week, the mice were sacrificed and the tumors were collected for analysis.

### Plasmid conduction and luciferase assay

The pMIR-report plasmid with the binding sequences of miR2911 and the mutants applied in this work was described previously [10]. For the luciferase reporter assays, 0.8 µg of the report plasmid, 0.8 g β-galactosidase expression vector (Ambion, Austin, TX, USA) and 80 pmol mature miR2911 or ncRNA were transfected into cells cultures in 6-well plates. After 24 h, the cells were analyzed using a luciferase assay kit (Promega, Madison, WI, USA).

### Enzyme linked immunosorbent assay (ELISA)

To validate whether miR2911 could down-regulate TGF-β1 expression, CT26 cells were plated into 6-well plates, and exposed to ncRNA or miR2911 (2 nmol/mL) in RPMI-1640 medium containing FBS. After 24 h, the supernatant was collected for TGF-β1 test using an ELISA kit (4A Biotech, Beijing, China), according to the manufacturer’s instructions. To determine the level of TGF-β1 in tumor tissues of *Sidt1*^+*/*+^ or *Sidt1*^*−/−*^ mice, five times volume of cold PBS homogenate was added and homogenated. Centrifuged at 6000 r/min for 15 min, the supernatants were collected. The supernatants were tested using a TGF-β1 ELISA kit from Biotech, following the kit’s instructions.

### Haematoxylin and eosin (H&E) staining

Tissues were fixed in 4% paraformaldehyde (PFA) over night, processed and embedded in paraffin. Sections (5 μm in sickness) were prepared and stained according to the manufacturer’s instructions (Jiancheng Bioengineering Institute, Nanjing, China). The stained sections were photographed under a BX51microscope (Olympus, Tokyo, Japan).

### Immunostaining

Tissues were embedded in OCT medium. Sections (5 μm in sickness) were mounted on poly-l-Lysine coated glass slides (Citotest, Haimen, China). The sections were blocked with 5% bovine serum albumin (BSA) for 1 h and then stained with primary antibody at 4 °C overnight. The sections were incubated with the corresponding fluorescent labeled secondary antibody (Thermo Fisher Scientific, Waltham, MA, USA) for 45 min at room temperature, followed by diamidine phenylindole (DAPI) staining for nuclei. Fluorescence was visualized under a Leical TCS SP8 confocal microscope (Leica Microsystems).

### Statistical analysis

All data were normally distributed. Data are shown as mean ± standard error of the mean (s.e.m.). The sample size (n) was set up according to the pre-experimental data. Student’s *t*-test, one-way and two-way analysis of variance (ANOVA) followed by Bonferroni’s multiple comparison test, and log-rank test were performed. A *P value < *0.05* was considered significant.

## Results

### Honeysuckle inhibited the development of CT26 tumors in vivo

**Fig. 1:**
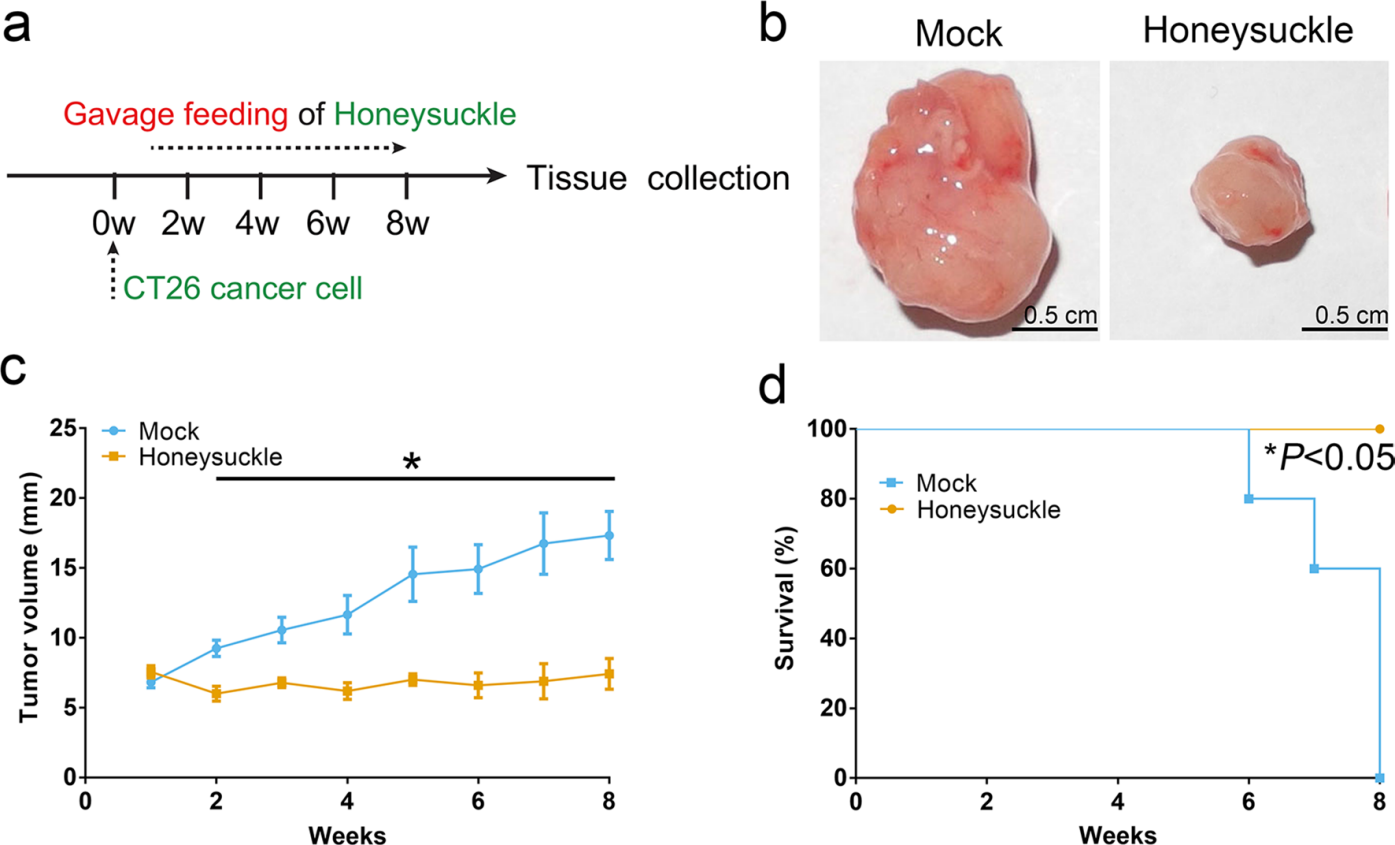
The anticancer effect of honeysuckle on CT26 tumors in vivo. **a** Schematic diagram of assessing the anticancer effect of honeysuckle after the implantation of CT26 cells. **b** Gross view of the tumors in the honeysuckle-treated group and the control group. **c** The average sizes of tumors of the mice with different treatments. **d** Survival rates of the mice with different treatments. Data represent mean ± s.e.m., *n* = 5 per group. *P < *0.05* was significant different between mock group and honeysuckle-treated group

As shown in Fig. 1a, we established a CT26 tumor-bearing C57BL/6 mice model to verify the inhibition effect of honeysuckle on colon cancer development in vivo. Honeysuckle was administered orally once daily 1 week after the tumor cells transplantation, and the width and length of the tumors were measured every week. The tumor size (7.42 mm in average diameter) of mice treated with honeysuckle smaller than that of control animals (17.33 mm) at week 8 (Fig. 1b, c). The mice with honeysuckle treatment all survived through the study period, while the mock-treated ones all died within 8 weeks after the cancer cells transplantation (Fig. 1d). In short, these results suggested that honeysuckle could effectively slow the development of CT26 tumors down.

### miR2911 was highly enriched in honeysuckle and had a high stability

**Fig. 2:**
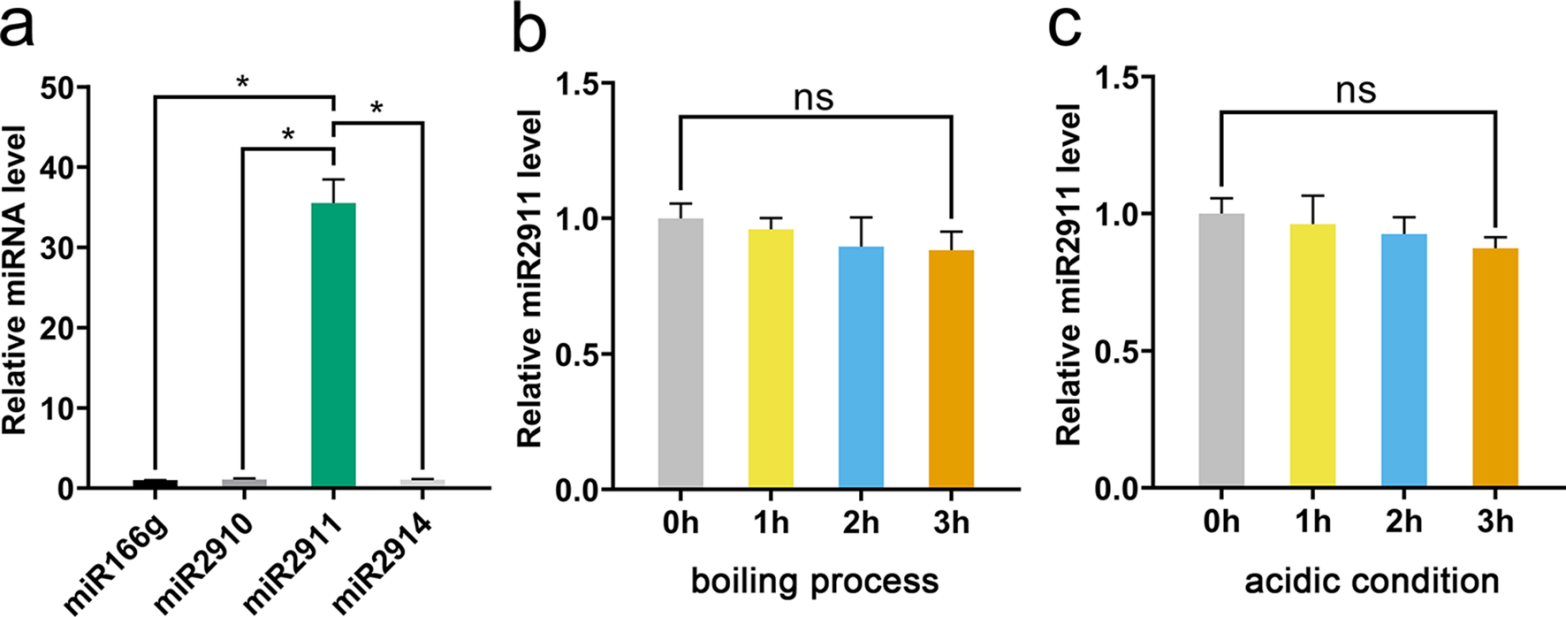
miR2911 is highly enriched in honeysuckle decotion and possesses a high stability. **a** The levels of microRNAs in honeysuckle decoction. **b** The miR2911 levels in honeysuckle decoction boiled for 0 h, 1 h, 2 h and 3 h. **c** The miR2911 levels in honeysuckle decoction acidified by HCl to pH 2.0 and incubated for 0 h, 1 h, 2 h and 3 h. Data represent mean ± s.e.m., *n* = 3 per group. *P < *0.05* was significant different

Compared with miR168a, miR172a, miR2915 and miR396a, miR166g, miR2910, miR2911 and miR2914 from honeysuckle displayed high content (Additional file 1: Figure S1). This find helped us identified controlled microRNAs for the stability analysis of miR2911. The level of expression of miR166g, miR2910, miR2911 and miR2914 from honeysuckle decoction were analyzed by qRT-PCR. Among these microRNAs, miR2911 enriched in honeysuckle represented the highest amount (Fig. 2a). To examine the stable existence of miR2911, we treated honeysuckle decoction in boiling process and acidic condition for 0 h, 1 h, 2 h and 3 h. As shown in Fig. 2b, c, boiling and acidification did not significantly affect the quality of miR2911, suggesting the high stability of miR2911.

### Honeysuckle-derived miR2911 targeted TGF-β1 mRNA and reduced the factor expression

**Fig. 3:**
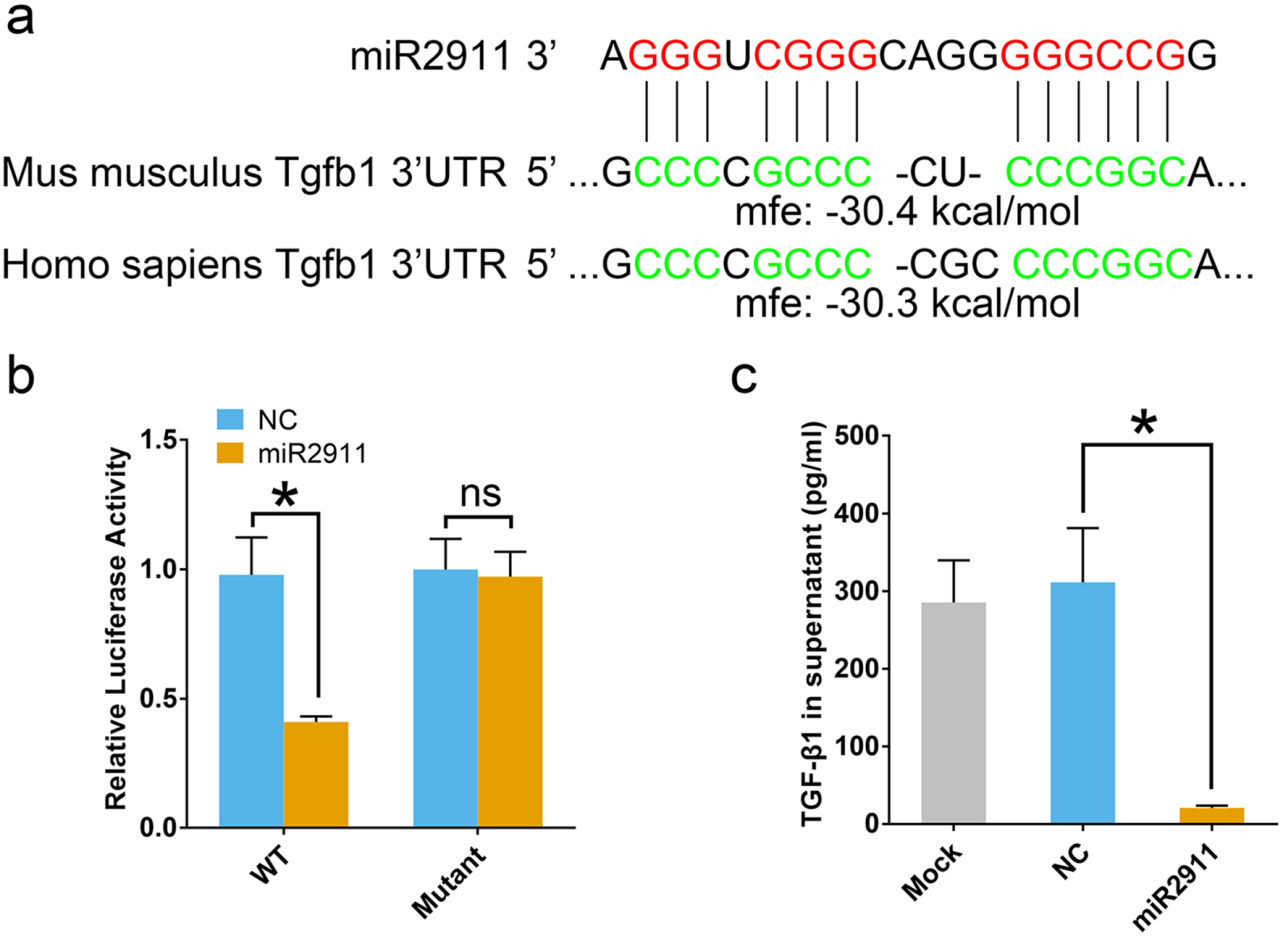
Identification of TGF-β1 as a target of miR2911 in vitro. **a** Schematic description of the base pairing between miR2911 and TGF-β1 mRNA (mouse and human), and their binding energies. **b** Luciferase activity of HEK293T cells co-transfected with firefly luciferase reporters with miR2911 or NC microRNA treatment. *WT* wild type firefly luciferase reporters with normal TGF-β1, *Mutant* firefly luciferase reporters with mutant TGF-β1 3′-UTR. **c** The expression levels of TGF-β1 of CT26 cells in the mock group, the ncRNA-treated group and the miR2911-treated group. Data represent mean ± s.e.m., *n* = 3 per group. *P < *0.05* was significant different between ncRNA-treated group and miR2911-treated group

Theoretically, honeysuckle-derived miR2911 could down-regulated TGF-β1 expression due to the ability to strongly bind to TGF-β1 (the binding energy between miR2911 and Mus musculus/Homo sapiens TGF-β1 are all lower than − 30 kcal/mol, Fig. 3a). To verify that TGF-β1 mRNA was a target of miR2911, we expressed a luciferase reporter in recipient HEK293T cells. Compared with ncRNA, miR2911 reduced luciferase activity to 41% (Fig. 3b). Meanwhile, the reduction was abolished with miR2911-binding site mutation. To estimate whether miR2911 down-regulated TGF-β1 expression, we treated CT26 cells with miR2911 and then found a significantly lower expression level of TGF-β1 in the supernatant than the mock-treated one (Fig. 3c). The above data demonstrated that honeysuckle-derived miR2911 could bind to TGF-β1 gene and inhibited its translation.

### SIDT1 helped miR2911 to perform the down-regulation of TGF-β1

**Fig. 4:**
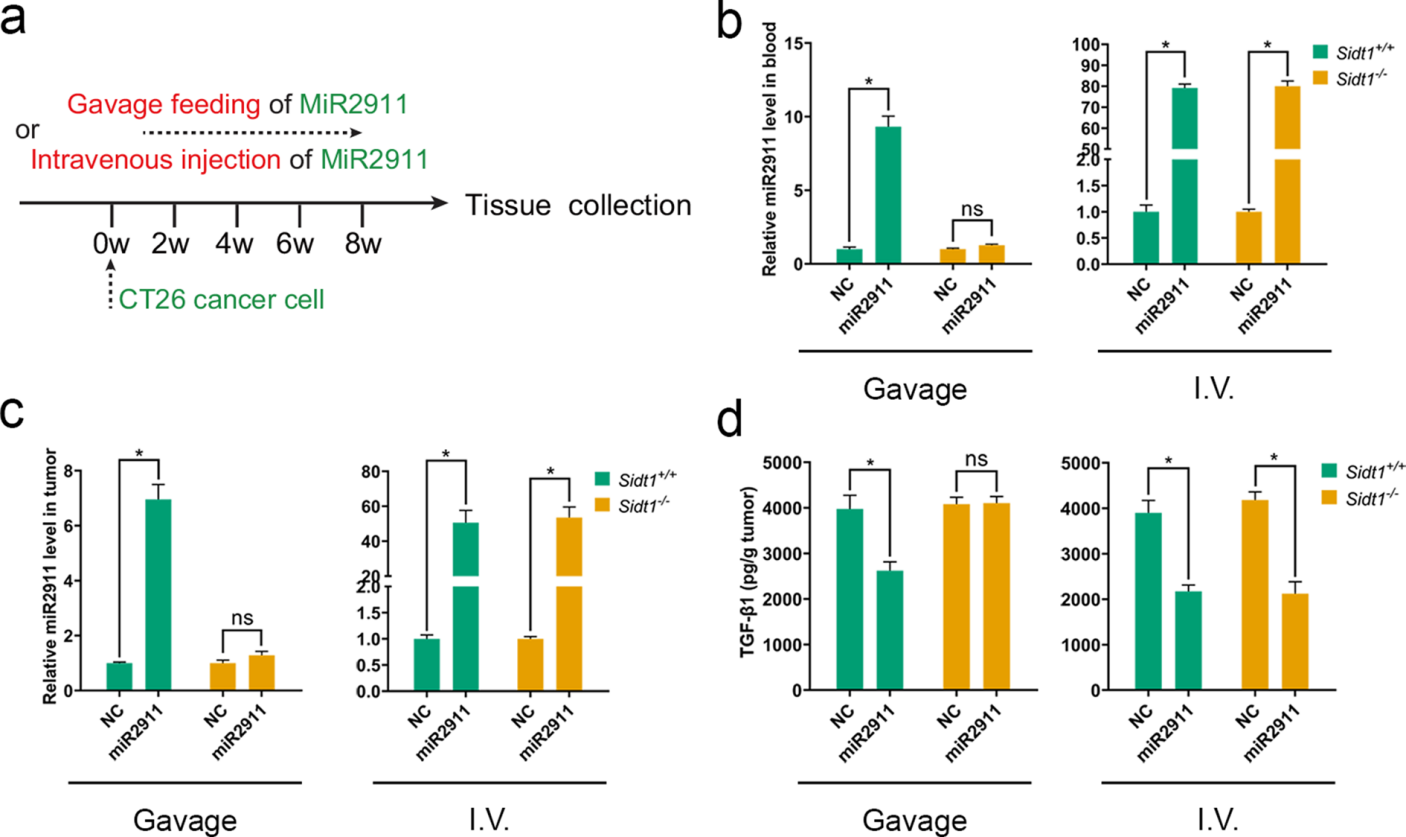
SIDT1 helped miR2911 to transfer between species and realize the down-regulation of TGF-β1. **a** Diagram of miR2911 gavage feeding or intravenous injection treatment after the implantation of CT26 cells. **b** The miR2911 levels in blood of *Sidt*^+*/*+^ or *Sidt*^*−/−*^ mice after gavage feeding or intravenous injection. **c** The miR2911, **d** TGF-β1 levels in CT26 tumor of *Sidt1*^+*/*+^ or *Sidt1*^*−/−*^ mice after gavage feeding or intravenous injection. Data represent mean ± s.e.m., *n* = 5 per group. *P < *0.05* was significant different

Dietary miR2911 absorbed via SIDT1 are secreted and inhibited CCl_4_-induced liver fibrosis in *Sidt1*^+*/*+^ mice, but failed in *Sidt1*^*−/−*^ mice, suggesting that miR2911 absorbed via SIDT1 secreted and played biological roles in vivo [14]. To examine the effect of SIDT1 on miR2911, we adopted a CT26 tumor model in *Sidt1*^+*/*+^ and *Sidt1*^*−/−*^ mice. As shown in Fig. 4a, miR2911 was administered intravenous (I.V.) injection or orally once daily 1 week after the tumor cells transplantation. In the gavage groups, dramatically increased miR2911 levels were detected in the blood and tumor of *Sidt1*^+*/*+^ mice (Fig. 4b, c), and consequently, the expression of TGF-β1 was significantly suppressed (Fig. 4d). However, in the *Sidt1*^*−/−*^ mice, the increase in miR2911 and suppression in TGF-β1 expression were abolished (Fig. 4b–d). The above results demonstrated that the SIDT1 helped miR2911 to perform function and down-regulate TGF-β1.

### Honeysuckle-derived miR2911 inhibited the progression of CT26 tumors in vivo

**Fig. 5:**
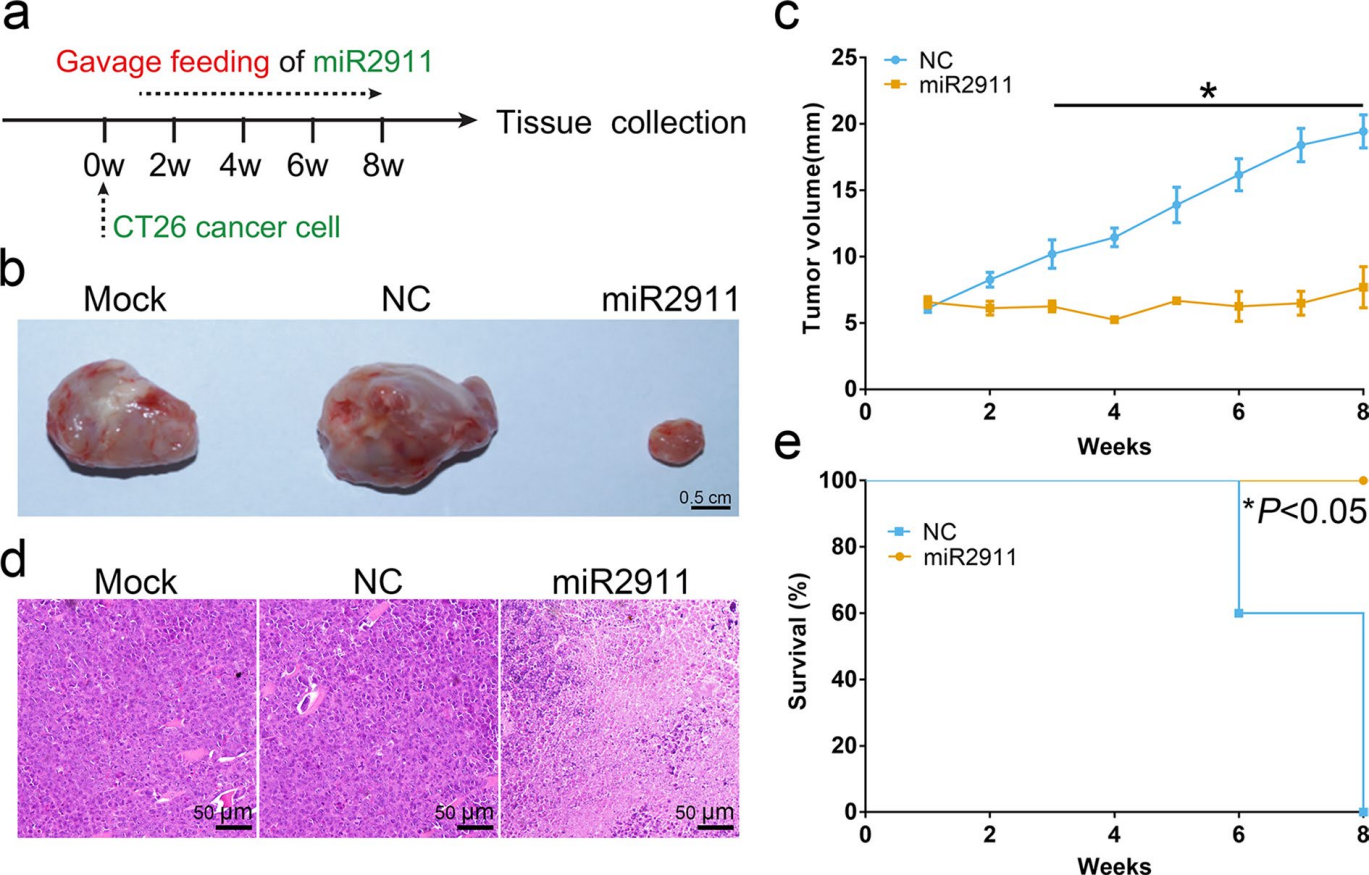
The anticancer effect of miR2911 in vivo. **a** Schematic diagram of assessing the anticancer effect of honeysuckle after the implantation of CT26 cells. **b** Gross view of the tumors in mock group and miR2911-treated group. **c** The tumor sizes of the mice with different treatments. **d** Representative photographs of the colon tumors with H&E staining. **e** Survival rates of the mice with different treatments. Data represent mean ± s.e.m., *n* = 5 per group. *P < *0.05* was significant different between ncRNA-treated group and miR2911-treated group

Previous studies showed that TGF-β1 was a key tumor-promoting factor in immunocompetent mice [24]. The above results proved that honeysuckle-derived miR2911 targeted TGF-β1 gene, thereby decreased the expression of TGF-β1 protein. Therefore, combining the above evidence, we assumed that miR2911 was a vital anti-tumor component of the magical herb. To validate whether miR2911 could inhibit colon tumor development, we established a CT26 tumor-bearing immunocompetent mice model. miR2911 was administered orally once daily 1 week after the tumor cells transplantation (Fig. 5a). At week 8, we sacrificed the mice and collected the tumor tissues for analysis. The average tumor size of the miR2911-treated group was markedly decreased (7.00 mm in average diameter) relative to that of the ncRNA-treated group (19.45 mm, Fig. 5b, c). Further histological analysis of the tumor tissues was performed. Crowds of necrotic tumor cells were observed in the miR2911-treated group. However, the phenomenon was rarely observed in the ncRNA-treated group (Fig. 5d). Meanwhile, the tumor-bearing mice treated with miR2911 had significantly better survival than the control ones (Fig. 5e). Similar to honeysuckle, miR2911 slowed the colon cancer progress down, suggesting that miR2911 was an active anticancer component of honeysuckle.

### T cells played a key role in tumor suppressor effects of miR2911

**Fig. 6:**
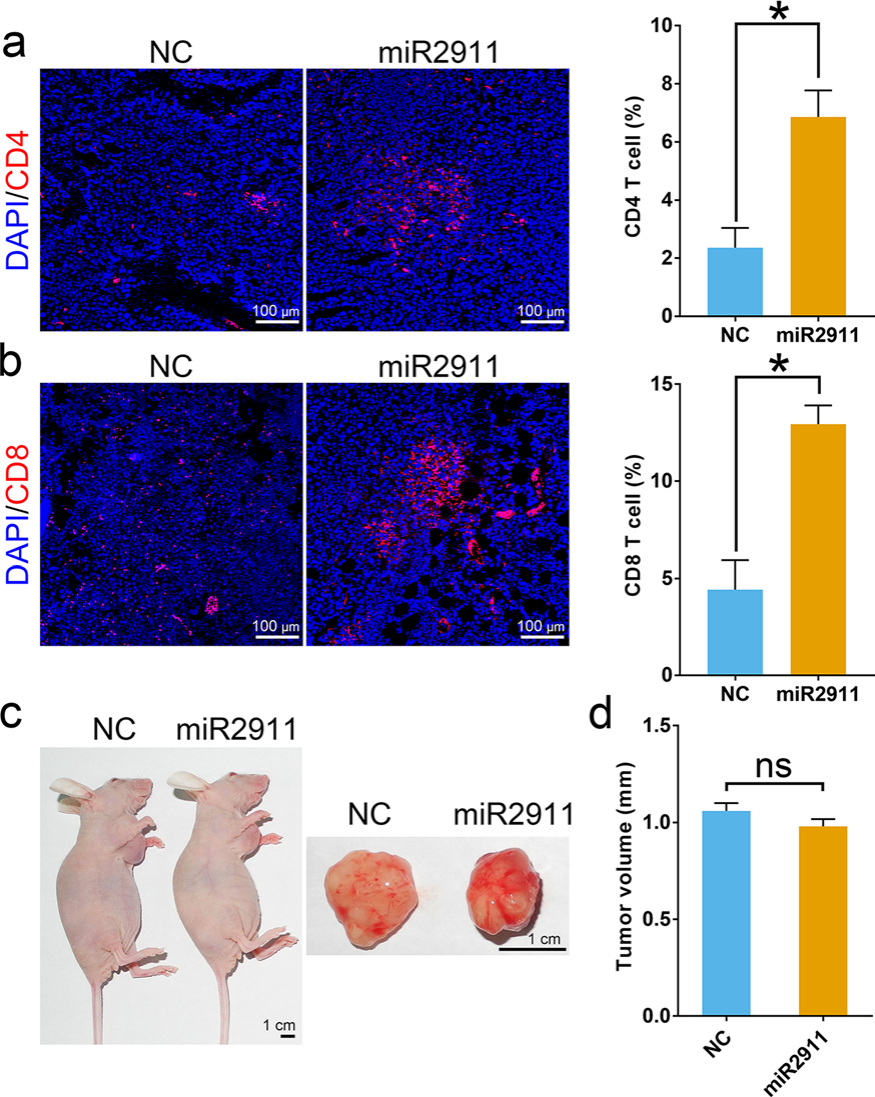
The role of T cells in the anti-tumor effect of miR2911. **a**, **b** Representative immunostaining images for CD4/CD8 immunostaining of the tumors in the ncRNA-treated group and the miR2911-treated group, and the quantitative measurement of positive/negetive area percentage. **c** Gross view of the tumor-bearing nude mice treated with ncRNA and miR2911, and the corresponding tumors. **d** The tumor sizes from the nude mice after different treatments. Data represent mean ± s.e.m., *n* = 5 per group. *P < *0.05* was significant different between ncRNA-treated group and miR2911-treated group

Based on the results mentioned above, we confirmed that honeysuckle-derived miR2911 could inhibit colon tumor development by targeting TGF-β1 mRNA. Previous studies revealed that TGF-β1 could inhibit infiltration of T lymphocytes in tumors [21–23]. It is, therefore, reasonable to wonder if miR2911 exerted its anti-tumor activity by increasing the infiltration of T lymphocytes in tumors. As illustrated in Fig. 6a, b, compared with that of the ncRNA-treated group, the tumors in the miR2911-treated group showed a higher percentage of T cells (CD4^+^ T cells: 2.36 vs. 6.86 percentage, and CD8^+^ T cells: 4.42 vs. 12.94 percentage). These results indicated that miR2911 treatment indeed increased numbers of T cells in tumors. To further analyze in tumor suppressor effects of miR2911, we established the tumor-bearing nude mice models, in which T cells cannot mature and thus are unable to exert their function. The average size of tumors did not differ between the miR2911-treated group and the control group. (Fig. 6c, d). This implied that loss of function of T cells blocked the anti-tumor effect of microRNA. Taken together, these results revealed that miR2911 relied on functioning T cells to exert its anti-tumor effect.

## Discussion

In the present study, we found that honeysuckle-derived miR2911 could target TGF-β1 and inhibit colon tumor development in immunocompetent mice. Meanwhile, the anti-tumor effect was abolished in immune deficiency mice with loss function of T cells.

Compared with other microRNAs in honeysuckle, miR2911 possesses high stability during the boiling process and unique TGF-β1 targeting [14]. A recent study has proved that miR2911 absorbed via SIDT1 transporter suppressed the expression of TGF-β1, resulting in alleviation of CCl_4_-induced liver fibrosis [14]. TGF-β1 not only participates in the development of liver fibrosis, but also promotes tumor progression. Our research showed that honeysuckle-derived miR2911 decreased TGF-β1 secretion from colon cancer CT26 cells and increased numbers of T lymphocytes in CT26 tumors, finally resulting in the inhibition of cancer progression. Despite the absence of clear evidence that miR2911 specific siRNA could abolish the anti-tumor effect of honeysuckle to prove the crucial role of miR2911 in the anticancer effect of honeysuckle, we have shown that honeysuckle-derived miR2911 was the active anti-tumor component.

Additionally, we explored the anticancer mechanism of miR2911. miR2911 strongly bind to TGF-β1 gene leading to a decrease of the expression of its protein. This resulted in an increased number of T cells in the tumor, which ultimately led to the inhibition of tumor development. However, the function exertion of TGF-β1 is a complex process, including mRNA and protein expression, latent TGF-β1 activation, as well as the binding to its receptor [25, 26]. In theory, miR2911 down-regulates the TGF-β1 expression at the transcription level. In our present study, miR2911 treatment led to a decrease of TGF-β1 secretion. However, it has been difficult to figure out exactly how much these secreted TGF-β1 was activated, and how much activated TGF-β1 binds to its receptor. In our view, miR2911 could firmly bind to TGF-β1 gene-this was strongly demonstrated by their extremely low binding energy. This results in a low level of TGF-β1 that is far below the level to maintain immune suppressive effect during cancer development. The disruption of the immune suppressive effects finally leads to the inhibition of cancer development.

We found that miR2911 targeted TGF-β1, and then broke the cancer-promoting effect of TGF-β1, finally led to slowing the cancer progression down. In addition to miR2911, honeysuckle contains various microRNAs, such as miR166g, miR2914, and miR2910. Whether other microRNAs participate in the anti-tumor effect remains unknown. Therefore, the role of other microRNAs of honeysuckle in the anti-tumor effect is worthy of further study. Apart from microRNAs, many kinds of polysaccharides, proteins, and other small molecules present in honeysuckle. Whether a positive interaction mechanism among these components in honeysuckle is present is currently not well understood.

Since miR2911 exerted anti-cancer function by regulating immune cells, we further predicted the effects of miR2911 on major immune checkpoint genes (Additional file 1: Figure S2). Strikingly, honeysuckle-derived miR2911 could strongly bind to PD-1, PDL-1, and CTLA-4. The result enlightened us that miR2911 may be a natural cancer immunotherapy medicine.

## Conclusion

This study has shown that the honeysuckle-derived miR2911 is an active anti-tumor component. miR2911 targeted TGF-β1 and reversed the cancer-promoting effect of TGF-β1 via increasing numbers of T lymphocytes in tumors, finally led to the inhibition of tumor development.

## Supplementary Information

**Additional file 1: Figure S1.** The content of microRNAs in honeysuckle. **a** The photo of honeysuckle. **b **The levels of plant microRNAs in honeysuckle. **Figure S2.** Prediction of the major immune checkpoint genes sequence targeted by miR2911. **a–c** Schematic description of the base pairing between miR2911 and major immune checkpoint genes (PD-1, PDL-1 and CTLA4; mouse and human), and their binding energies.

## Data Availability

The datasets used and/or analyzed during the current study are available from the corresponding author on reasonable request.

## Abbreviations

BSA: Bovine serum albumin
CT26: Colorectal adenocarcinoma cells
ELISA: Enzyme linked immunosorbent assay
FBS: Fetal bovine serum
H&E: Haematoxylin and eosin
PFA: Paraformaldehyde
qRT-PCR: Quantitative real-time polymerase chain reaction
RPMI-1640: Roswell Park Memorial Institute-1640 medium
SIDT1: SID-1 transmembrane family member 1
SPF: Specific pathogen-free
TGF-β1: Transforming growth factor-β1
