# Supplementary material for: Honeysuckle-derived microRNA2911 inhibits tumor growth by targeting TGF-β1
Source: Chin Med. 2021 Jun 29;16:49. doi: 10.1186/s13020-021-00453-y (PMC8244210; doi:10.1186/s13020-021-00453-y)
Supplement: Supplementary file 1 — Additional file 1: Figure S1. The content of microRNAs in honeysuckle. a The photo of honeysuckle. b The levels of plant microRNAs in honeysuckle. Figure S2. Prediction of the major immune checkpoint genes sequence targeted by miR2911. a–c Schematic description of the base pairing between miR2911 and major immune checkpoint genes (PD-1, PDL-1 and CTLA4; mouse and human), and their binding energies. [file 13020_2021_453_MOESM1_ESM.pdf]

Supplementary material

Honeysuckle-derived microRNA2911 inhibits tumor growth by targeting TGF-β1

Chunyan Liu[1], Mengzhen Xu[1], Luocheng Yan[1], Yulian Wang[1], Zhen Zhou[1], Shaocong Wang[1], Yajie Sun[1], Junfeng Zhang[1*] and Lei Dong[1*]

[1]State Key Laboratory of Pharmaceutical Biotechnology, School of Life Sciences, Nanjing University, 163 Xianlin Avenue, Nanjing 210093, China.

*Correspondence to: L. Dong, Email: leidong@nju.edu.cn; J. Zhang, Email: jfzhang@nju.edu.cn.

Full list of author information is available at the end of the article.

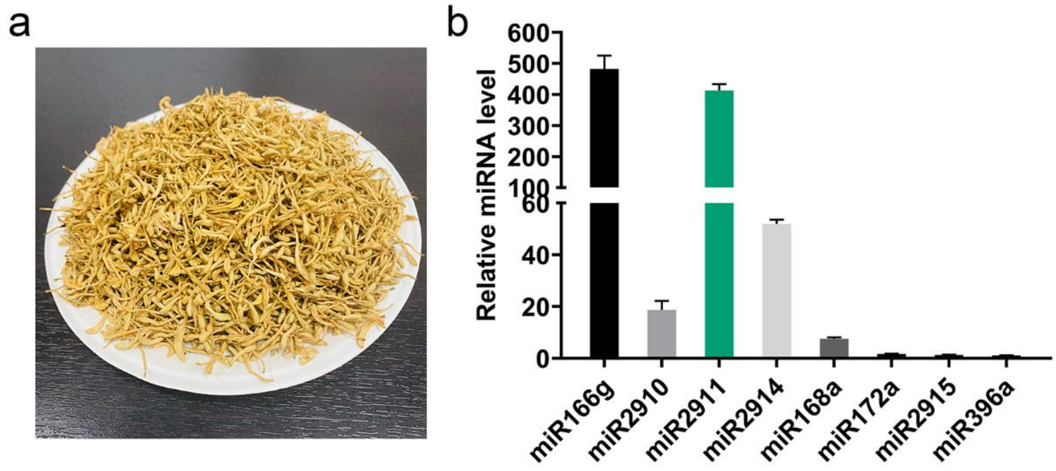

Fig. S1 a The photo of honeysuckle. **b** The levels of plant microRNAs in honeysuckle.

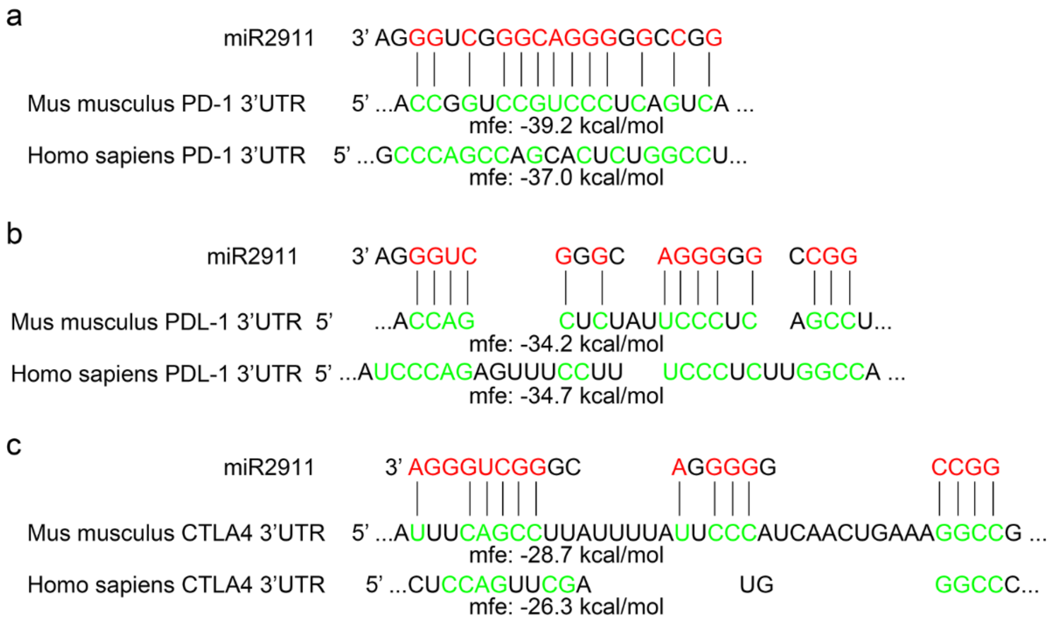

Fig. S2 a-c Schematic description of the base pairing between miR2911 and major immune checkpoint genes (PD-1, PDL-1 and CTLA4; mouse and human), and their binding energies.
